# TLR4 drives the pathogenesis of acquired cholesteatoma by promoting local inflammation and bone destruction

**DOI:** 10.1038/srep16683

**Published:** 2015-12-07

**Authors:** Yu Si, Yu Bin Chen, Sui Jun Chen, Yi Qing Zheng, Xiang Liu, Yi Liu, Huai Li Jiang, Guo Xu, Zhuo Hao Li, Qiu Hong Huang, Hao Xiong, Zhi Gang Zhang

**Affiliations:** 1 Department of Otolaryngology Head and Neck Surgery, Sun Yat-Sen Memorial Hospital, Sun Yat-Sen University, Guangzhou, 510120. China; 2 Institute of Hearing and Speech-Language Science, Sun Yat-sen University, Guangzhou, 510120. China; 3 Department of Otolaryngology Head and Neck Surgery, The third affiliated Hospital, Sun Yat-Sen University, Guangzhou, 510630. China

## Abstract

Acquired cholesteatoma is a chronic inflammatory disease characterized by both hyperkeratinized squamous epithelial overgrowth and bone destruction. Toll-like receptor (TLR) activation and subsequent inflammatory cytokine production are closely associated with inflammatory bone disease. However, the expression and function of TLRs in cholesteatoma remain unclear.We observed inflammatory cell infiltration of the matrix and prematrix of human acquired cholesteatoma, as well as dramatically increased expression of TLR4 and the pro-inflammatory cytokines TNF-α and IL-1β. TLR2 exhibited an up-regulation that was not statistically significant. TLR4 expression in human acquired cholesteatoma correlated with disease severity; the number of TLR4-positive cells increased with an increased degree of cholesteatoma, invasion, bone destruction, and hearing loss. Moreover, TLR4 deficiency was protective against experimental acquired cholesteatoma-driven bone destruction and hearing loss, as it reduced local TNF-α and IL-1β expression and impaired osteoclast formation by decreasing expression of the osteoclast effectors receptor activator of nuclear factor (NF)-κB ligand (RANKL) and tartrate-resistant acid phosphatase (TRAP). TLR2 deficiency did not relieve disease severity, inflammatory responses, or osteoclast formation. Moreover, neither TLR2 nor TLR4 deficiency had an effect on antimicrobial peptides, inducible iNOS,BD-2 expression or bacterial clearance. Therefore, TLR4 may promote cholesteatoma-induced bone destruction and deafness by enhancing inflammatory responses and osteoclastogenesis.

Cholesteatomas are usually classified as congenital or acquired. Congenital cholesteatoma is classically defined as an epithelial inclusion behind an intact tympanic membrane without a history of otitis media. Often congenital cholesteatoma is asymptomatic and is discovered during a routine ear examination^1^. Acquired cholesteatoma is a chronic inflammatory disease characterized by both the overgrowth of hyperkeratinized squamous epithelium and bone erosion in the middle ear, and it is a major cause of deafness^2^. The annual incidence of acquired cholesteatoma is upto 9.2 per 10,000 individuals^3^. Acquired cholesteatomas are very aggressive and gradually expand. They ultimately cause complications because of the erosion of adjacent bony structures, resulting in destruction of the ossicular chain and otic capsule and subsequent hearing loss, vestibular dysfunction, facial paralysis, and intracranial complications. The only effective intervention is tympanomastoid surgery to remove the lesion^4^. However, surgery cannot resolve the bone loss or prevent recurrence, so more than 70% of patients require reoperation during a 10-year follow-up period. Moreover, repeated operations can increase hearing damage^5^. Indeed, the poor treatment efficacy and the inability to reverse bone loss highlight the need to identify novel targets to improve therapeutic efficacy and patient outcomes.

Nearly all acquired cholesteatomas are chronically infected; 85% of acquired cholesteatomas contain bacteria, most commonly *Pseudomonas aeruginosa* (PA)^6^. As detectedby clinical observation, infected cholesteatomas tend to become more rapidly enlarged and to destroy local structures^7^.Upon becoming super-infected, congenital cholesteatomas may expand, resulting in bone destruction and chronic ear infection, similar to those caused by acquired cholesteatoma^8^. Therefore, bacterial infection may enhance the aggressiveness of cholesteatoma, but the mechanisms involved remain unclear.

The mucosal innate immune system, characterized by epithelial and other mucosal cells, actively participates in the host response to bacterial infection^9^. This first line of defense is triggered through the recognition of pathogens by Toll-like receptors (TLRs) and the subsequent expression and secretion of pro-inflammatory cytokines^10^,^11^.TLRs are a family of pathogen-associated molecular pattern recognition receptors that are key mediators of the recognition of pathogens by the innate immune system^12^.TLRs initiate the innate immune response, which ultimately involves inflammatory cell infiltration, inflammatory cytokine production, and defense against bacterial infection^13^. Bones are dynamic organs that are constantly remodeled to achieve both calcium homeostasis and structural integrity. Matrix synthesis is carried out by osteoblasts, whereas resorption is exclusively performed by osteoclasts^14^. Under normal physiological conditions, these activities are carefully balanced; however, as much as 10% of the total bone content in an adult human is replaced annually^15^. However, bacterial infection and subsequent inflammatory responses disrupt this balance via overactive osteoclasts, which can lead to bone destruction^16^. Chronically inflamed tissues adjacent to the bone can be observed in many diseases, and this chronic inflammation is capable of eventually causing bone resorption. In rheumatoid arthritis, the chronically inflamed pannus erodes the adjacent bone and cartilage, thereby causing debilitating joint disease^17^. In the oral cavity, chronic inflammation caused by gingivitis can result in erosion and loosening of the adjacent bone^18^.

Acquired cholesteatomas are characterized by the increased adherence of bacteria to entrapped keratin in addition to keratinocyte proliferation; these processes result in a “matrix” of desquamated keratinocytes that form an expanding mass^19^. In the past decade, conclusive evidence that bone resorption resulting from cholesteatoma is the sole consequence of osteoclastic resorption has been reported^20^. TLR activation and subsequent inflammatory cytokine production play key roles in osteoclast formation^21^. However, few studies onthe role of TLRs in cholesteatoma-induced bone destruction have been conducted. Therefore, we first performed histological analyses of congenital and acquired cholesteatomas from patients. Next, we assessed the expression of TLRs and downstream cytokines to evaluate whether TLR4 is associated with acquired cholesteatoma-induced bone resorption. Furthermore, we established experimental cholesteatoma models to verify the manner in which TLR4 promotes acquired cholesteatoma-induced bone destruction.

## Results

### Human congenital and acquired cholesteatomas have qualitatively distinct histopathologies

**Figure 1:**
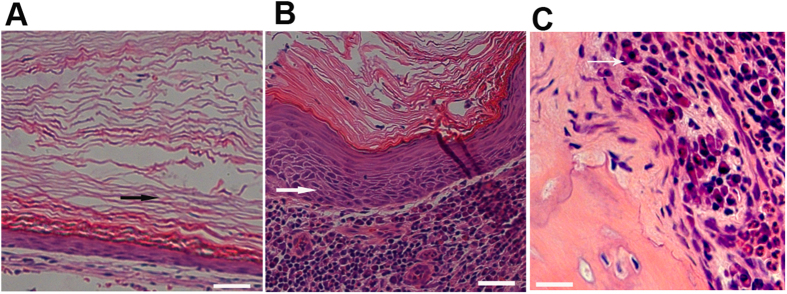
Pathological profiles of congenital versus acquired cholesteatoma and eroded bone. Representative serial sections of cholesteatomas from CC and AC patients were used to analyze pathological profiles (n = 12). Congenital cholesteatoma (**A**) acquired cholesteatoma (**B**) and eroded bone from acquired cholesteatoma patients (**C**) were stained with hematoxylin and eosin, as described in the Materials and Methods section. The images in panels (**A–C**) were obtained using a 20× objective. Black arrows indicate lamellar sheets of keratin and an incrassated epithelium, and the large black asterisk in (**C**) indicates an osteoclast.

To identify biological characteristics that differ between congenital and acquired cholesteatomas, we assessed the pathological profiles of each condition. Congenital cholesteatomas appeared as lamellar sheets of keratin overlaying a thin squamous epithelium without inflammatory cells (Fig. 1A). In contrast, acquired cholesteatomas presented as lamellar sheets of keratin along an incrassated and inflamed epithelium with lymphocyte and polymorphonuclear cell infiltration of the prematrix (Fig. 1B). Furthermore, osteoclasts were present at the ossicles that had been eroded by acquired cholesteatomas (Fig. 1C). These data imply that infection and inflammation caused by human acquired cholesteatomas may lead to different biological characteristics compared with congenital cholesteatomas.

### Increased TLR4 and pro-inflammatory cytokine expression in human acquired cholesteatomas

**Figure 2:**
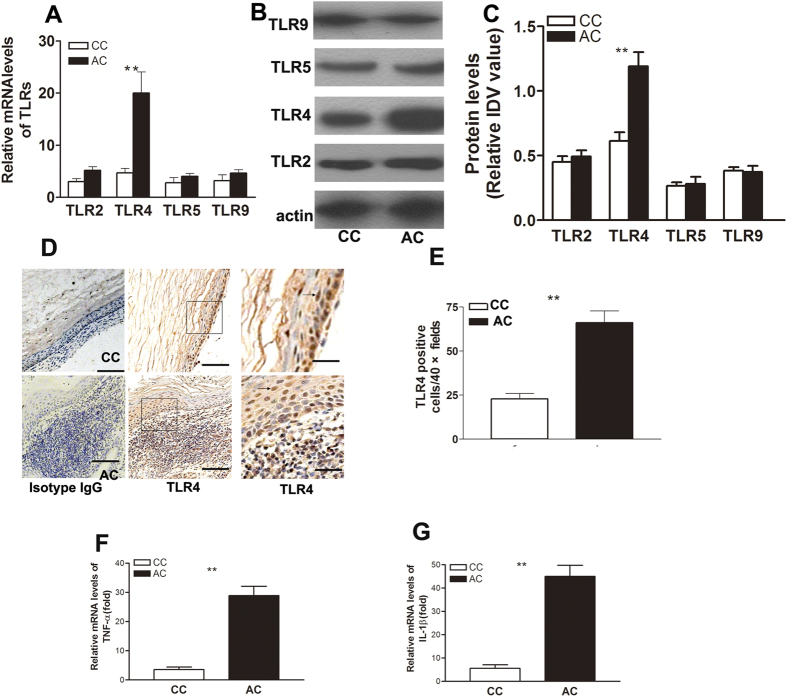
TLR4 and pro-inflammatory cytokines are up-regulated in human acquired cholesteatoma. (**A**) TLR2, TLR4, TLR5, and TLR9 mRNA levels were measured in CC and AC patients using real-time PCR. The relative mRNA expression levels were calculated after normalization to those of β-actin. Data indicate the mean ± SEM of three individual experiments (n = 16 patients per experiment). The TLR4 mRNA transcript levels were significantly up-regulated in acquired cholesteatoma patients. (**B**) TLR protein levels were further examined using Western blotting. An equal amount of protein (20 μg) was loaded in each lane. Western blot data represent one of three individual experiments using 7–8 pooled mucosal samples/run, and individual experiments were carried out in triplicate (CC, n = 7; AC, n = 8). (**C**) Band intensity was quantitated and normalized to that of the β-actin control, TLR4 protein was the most increased in AC. (**D–E**) Representative images of immunohistochemical staining for TLR4 in CC and AC (n = 12).Lamellar sheets were keratin, and the brown cells were TLR4-positive.The number of TLR4-positive cells was much higher in AC. (**F–G**) The pro-inflammatory cytokines IL-1β and TNF-α were significantly up-regulated in AC compared with CC.Data represent the mean ± SEM from three independent experiments (n = 16 patients/experiment); CC: congenital cholesteatoma; AC: acquired cholesteatoma; **indicates P < 0.01.

Nearly all human acquired cholesteatomasinvolve bacterial infections, whereas congenital cholesteatomas are aseptic. TLR mediates increased pro-inflammatory responses and bone destruction in response to bacterial infections. To determine whether TLRs participate in the pathogenesis of human acquired cholesteatoma, we performed quantitative reverse transcription-polymerase chain reaction (RT-PCR) to screen a panel of TLRs related to bacterial infection in human congenital and acquired cholesteatomas. Among all TLRs that we tested,TLR4 was the most abundantly expressed in acquired cholesteatoma, followed by TLR2 (Fig. 2A). To assess TLR expression at the protein level, Western blotting was carried out and also showed that TLR4 was the most increased TLR in acquired cholesteatoma.The mRNA and protein levels of TLR2 were up-regulated, but this trend did not reach our threshold for significance (Fig. 2A–C). To identify the cellular source of TLR4 expression,immunohistochemic alanalysis was performed. We found that the number of TLR4-positive cells was much higher in human acquired cholesteatoma. TLR4-positive cells were mainly scattered among the inflammatory cells in the prematrix of acquired cholesteatoma tissues, and TLR4-positive cells in epithelial tissue of acquired cholesteatoma and congenital cholesteatoma showed no significant differences (Fig. 2D,E). Moreover, the pro-inflammatory cytokines tumor necrosis factor (TNF)-α and IL-1β were up-regulated in acquired cholesteatoma (Fig. 2F,G). These data indicate that inflammation in acquired cholesteatoma may be mediate by TLR4.

### TLR4 expression in human acquired cholesteatoma correlates with disease severity

To further explore the role of TLR4 in the pathogenesis of human acquired cholesteatoma, immunohistochemical analysis of TLR4 expression was performed in187 cases of human acquired cholesteatoma. We correlated TLR4-positive cell counts with the clinical characteristics of acquired cholesteatoma patients (Table 1). The number of TLR4-positive cells increased with an increased degree of cholesteatoma invasion, bone destruction, and hearing loss. However, no difference was observed in TLR4-positive cell counts according to disease duration, sex, or age. These data may indicate that TLR4 may promote the pathogenesis of acquired cholesteatoma to cause more destruction.

### TLR4 deficiency is protective against experimental acquired cholesteatoma derived bone destruction and hearing loss

**Figure 3:**
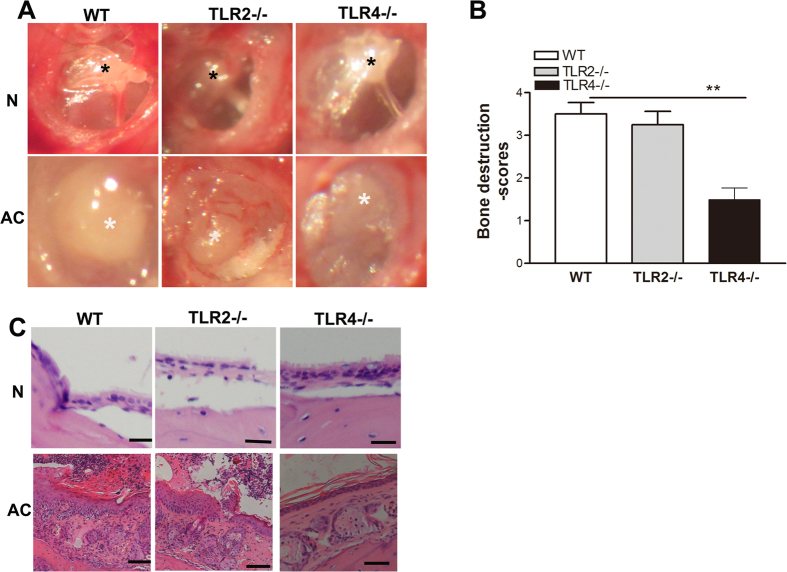
TLR4 deficiency results in diminished experimental acquired cholesteatoma-derived bone destruction and hearing loss. (**A**) The progression of experimental cholesteatoma, as detected by anatomical microscopy. WT,TLR2−/−, and TLR4−/− mice showed normal tympanic membraneswith clearly visible ossicles through the smooth, transparent tympanic membrane. At 6 weeks postoperatively, the tympanic membrane thickened in the pars flaccida, with cholesteatoma formation behind the tympanic membrane (n = 13). Black asterisks indicatethe malleus that could be observed through the smooth, transparent tympanic membrane, while white asterisks indicatecholesteatoma behind the tympanic membrane. (**B**) TLR4−/− mice showed markedly reduced bone destruction scores compared withWT mice (n = 13). (**C**) Representative images of bullae harvested fromWT, TLR2−/−,and TLR4−/− mice. Scale bars denote 200 μm (n = 8).

We used WT,TLR2−/−, and TLR4−/− mice to establish experimental models of cholesteatoma and evaluated the clinical features of these mice. The preoperative tympanicmembranes of WT, TLR2, and TLR4 mice were normal, with clearly visible ossicles through the smooth and transparent tympanic membrane. All mice developed acquired cholesteatoma at 6 weeks postoperatively, which was detected behind the tympanic membrane (Fig. 3A). To further compare the location of and bone destruction associated with cholesteatoma, bone destruction scores were used to semi-quantitatively compare the difference.The bone destruction scores were lowest in the TLR4−/−mice, but there was no significant difference in the scores between the TLR2−/−and WT mice (Fig. 3B). Furthermore, the histological pathologies of the bullae were verified by hematoxylin and eosin staining. The bullae from WT mice were filled with an expansive growth of epithelium and inflammatory cells that were overlaid with lamellar sheets of keratin. TLR2−/− mice showed moderate growth of epithelium and keratin, and a large number of inflammatory cells infiltrated into the matrix, whereas bullae from TLR4−/− mice showed a less thickened epithelium, fewer sheets of keratin, and less inflammatory cell infiltration (Fig. 3C).To measure the severity of the pathology in the middle ear, three indices were used for semi-quantitative evaluations, as shown in Table 3. The pathology scores were lowest in the TLR4−/−mice, but there was no significant difference in the scores between the TLR2−/− and WT mice (p < 0.05). The hearing functions of WT,TLR2−/−, and TLR4−/− mice were normal and occurred at a similar level preoperatively; however, the TLR4-deficient mice showed minor hearing loss compared with the TLR2−/− and WT miceat 6 weeks postoperatively (Table 2).

### TLR4 deficiency reduces local pro-inflammatory cytokine expression but does not affect bacterial clearance

**Figure 4:**
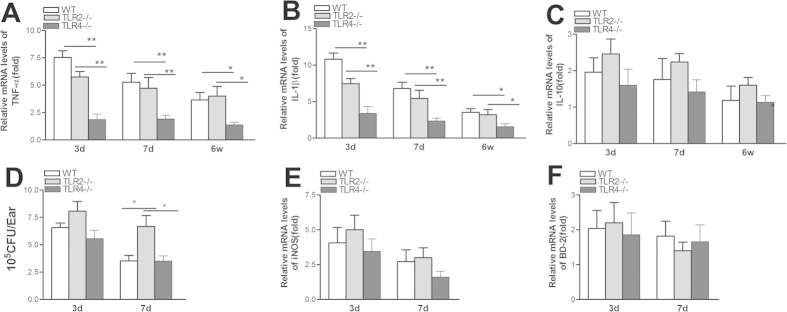
TLR4 deficiency reduces local pro-inflammatory cytokine expression but does not affect bacterial clearance. (**A–C**) TNF-α and IL-1β levels were significantly reduced in TLR4−/−mice compared with WT and TLR2−/− mice, while there was no significant difference between WT and TLR2−/− mice. IL-10 expression in the three mouse groups was similar at 3days,7days, and 6 weeks postoperatively. Bacterial loads (**C**) and antimicrobial peptide, iNOS, and BD-2 expression (**D–E**) were detected in the two groups based on bacterial plate counts and real-time PCR, respectively. Data are presented as the means ± SEM and represent two independent experiments, each with 5 animals per group.

The expression levels of pro- and anti-inflammatory cytokines (TNFα, IL-1β, and IL-10) measured in bulla extracts by qRT-PCR were compared between WT, TLR2−/−, and TLR4−/− mice at 3 days, 7 days, and 6 weeks postoperatively.TLR4 deficiency resulted in a significant decrease in the amounts of TNF-α (Fig. 4B) and IL-1β (Fig. 4C) mRNA levels compared with those in the WT and TLR2−/− mice, and there was no significant difference in the levels between the WT and TLR2−/− mice. IL-10 expression in the three mouse groups was similar. However,the bacterial loads and levels of antimicrobial peptide,iNOS,or BD-2 expression in the bullae were not significantly different between the WT, TLR2−/−, and TLR4−/− mice at 3 and 7 days postoperatively (p > 0.05). These data imply that TLR2 deficiency did not alleviate the inflammatory response to *Pseudomonas aeruginosa* infection and that TLR4 is a crucial modifier of acquired cholesteatoma that can trigger inflammation.

### TLR4 deficiency impairs osteoclast formation *in vivo*

**Figure 5:**
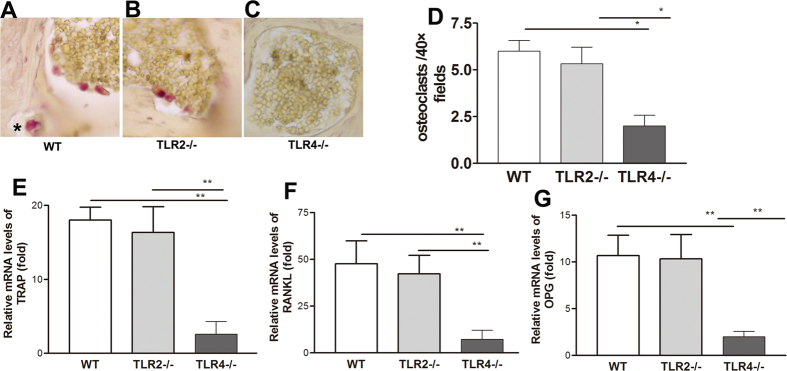
TLR4 deficiency reduces osteoclast formation *in vivo*. (**A–C**) Many TRAP-positive osteoclasts (arrows) were present in both WT and TLR2−/− mice but were largely absent in sections fromTLR4−/− mice. (**D–F**) TRAP, RANKL, and OPG expression was significantly reduced in TLR4−/− mice. Data indicatethe means ± SEM and represent two individual experiments with 5 animals per group per experiment.

We harvested bullae from WT, TLR2−/−, and TLR4−/− mice at 6 weeks postoperatively and analyzed them for the presence of osteoclasts by tartrate-resistant acid phosphatase (TRAP) staining. Large, multi-nucleated, TRAP-positive osteoclasts were observed within the prematrix of the cholesteatomathat invaded the bones of the WT and TLR2−/− mice, whereas TRAP-positive cells were generally absent in the TLR-4−/− mice (Fig. 5A–D). Furthermore, qRT-PCR analysis revealed a significant diminution of the local mRNA expression levels of TRAP inTLR4−/− mice compared with WT and TLR2−/− mice (Fig. 5E). Similarly, mRNA levels of the key osteoclast regulators receptor activator of nuclear factor (NF)-κB ligand (RANKL) and OPG were significantly reduced in the TLR4−/− mice (Fig. 5F,G).

## Discussion

The basic pathologies of congenital and acquired cholesteatomas are similar; specifically, a squamous epithelialline forms acyst, and progressive exfoliation of keratinous material into the interior of the cyst results in the production of a waxy white material that leads to slow expansion of the cyst^22^. Nearly all patients with acquired cholesteatomas suffer from inflammation and infection, which leads to the consequent formation of inflammatory granulation tissue with invading epithelia in acquired cholesteatomas^23^. Once the epithelium begins to hyperkeratinize, the destructive behavior of cholesteatomas is triggered^24^. In the present study, acquired cholesteatoma presented as an incrassated and inflamed epithelium and the infiltration of many inflammatory cells, which were mostly lymphocytes and polymorphonuclear cells, into the prematrix. Therefore, the inflammatory response in acquired cholesteatoma may result in aberrant behavior of the epithelium, resulting in aggressiveness and bone erosion.

The mucosal innate immune system, which is characterized by epithelial and other mucosal cells, actively participates in the host response to bacterial infection^9^. This first line of defense is triggered by the recognition of pathogens by pattern recognition receptors. Therefore, we screened a panel of TLRs related to bacterial infection in congenital and acquired cholesteatoma. Among all the TLRs that we tested, TLR4 but not TLR2 was up-regulated and related to the disease severity of human acquired cholesteatoma.TLRs recognize numerous structurally defined but not necessarily structurally related ligands. TLR3,TLR5,TLR7, TLR8, and TLR9 recognize “unique types” of ligands. Ligand discrimination by TLR2 and TLR4 is a complex process that is dependent on ligand compatibility, but it also requires the presence of specific co-receptors and accessory molecules^25^. In the case of TLR4, its best-characterized ligand is bacteriallipopolysaccharide (LPS). TLR2 is involved in sensing peptidoglycans, lipoteichoic acid, and lipoproteins expressed by Gram-positive bacteria^12^. *P.aeruginosa* is a Gram-negative bacterial pathogen that contains numerous PAMPs, including LPS, which is a toxic moiety that constitutes the major portion of the outermost membrane of Gram-negative bacteria^26^. Thus, the recognition of LPS via TLR4 is critical for host defense responses against infection by Gram-negative bacteria. However, recently published papers show that TLR2 but not TLR4 expression was significantly induced in response to the *P. aeruginosa*wt PAO1 strain. *P. aeruginosa*-dependent up-regulation of TLR2 influences the magnitude of proinflammatory responses to a secondary *S. aureus* infection^27^. To exclude the involvement of TLR2,we established a TLR2−/− mouse model of acquired cholesteatomato investigate the roll of TLR2 in acquired cholesteatoma. We found that TLR2 was up-regulated in human acquired cholesteatoma, but this trend did not reach our threshold for statistical significance. Moreover, TLR2 deficiency did not relieve disease severity, inflammatory responses, or osteoclast formation.Therefore, the pathogenesis of acquired cholesteatoma may be dependent on TLR4 instead of TLR2.

Previously, we successfully established an experimental cholesteatoma model in mice using a modified protocol for autologous metal skin graftimplantation plus *P. aeruginosa* infection. The clinical features of experimental cholesteatomas were evaluated by endoscopic and anatomical microscopic examinations, auditory brainstem response (ABR) threshold evaluations, and histological analysis, as previously reported^28^,^29^. TLR4 deficiency resulted in markedly reduced bone destruction scores and less hearing loss compared with those in TLR2−/− and WT mice. Therefore, TLR4 is required for acquired cholesteatoma-induced bone destruction, but the underlying mechanism still remains unclear.

Bacterial clearance and host inflammation play important roles in determining the outcomes of infection^30^. We showed that the host response, rather than bacterial survival, underlies resistance to bone resorption. We found areduced expression of the pro-inflammatory cytokinesTNF-α and IL-1β in the middle ear of TLR4-deficient mice compared with WT and TLR2−/− mice at 3days, 7days, and 6 weeks postoperatively, whereas bacterial loads and antimicrobial peptide,iNOS, and BD-2 expression in the bullae were not significantly different between the three groups of mice at 3 and 7 dayspostoperatively.The binding of LPS, the major component of *P. aeruginosa*, to TLR4 induces the production of the pro-inflammatory cytokines IL-1β, TNF-α, and IL-6 in macrophages, lymphocytes, and endothelial cells^31^,^32^, which is important for bone disease progression. Osteoclasts, multinucleated giant cells that resorb bone, develop from hemopoietic cells of the monocyte/macrophage lineage^33^,^34^. Bone marrow stromal cells have been shown to be involved in osteoclastogenesis. Indeed, osteoclastogenesis and osteoclast activation are primarily driven by the RANKL/RANK receptor signaling system and are tightly controlled and countered by OPG, the soluble decoy receptor for RANKL^35^,^36^.In the present study, we observed reduced RANKL and osteoclast formation in TLR4−/− mice. OPG functions as a soluble decoy-like factor for RANKL and, therefore, can act as a negative regulator of RANK signaling that is capable of inhibiting osteoclastogenesis *in vitro* and inducing osteoporosis when transgenically overexpressed in mice^37^. However, OPG was also unexpectedly decreased. The reduction in OPG may be due to the following reasons: OPG is a decoy for RANKL, as reduced RANKL may cause less activation of OPG,so the ratio of RANKL/OPG critically determines osteoclast formation;or there are other negative regulatory mechanisms of RANK signaling that have been described that inhibit osteoclastogenesis, such as IFN-β, which mediates a feedback mechanism that blocks further c-fos-dependent activity^38^. A more recently characterized negative regulatory mechanism of RANK signaling in OC precursor cells involves the formation of TRAF3-containing complexes on the RANK intracellular domain that inhibit both the canonical and non-canonical NF-κB pathways^39^.Ultimately, the degree of osteoclast differentiation and activation is dictated by the balance between OPG and RANKL within the bone microenvironment^40^. Herein,TLR4−/− mice displayed reduced RANKL and TRAP mRNA levels, which correlated with reduced osteoclast numbers. Additionally, TLR4−/− mice exhibited a down-regulation of inflammatory cytokines, such as TNFα, and IL-1β, that synergize with RANKL to promote osteoclast differentiation and activation^41^,^42^. TNF-α directly stimulates osteoclast differentiation, and fibroblast stimulation with IL-1β can also exert long-lasting effects on osteoclastogenesis^43^. Moreover, cytokines such as IL-1β, TNF-α, and RANKL can enhance the survival of osteoclasts^44^,^45^. Thus, TLR4 promotes pathology-induced bone destruction and deafness by enhancing inflammatory responses and osteoclastogenesis.

## Materials and Methods

### Ethics Statement

The procedures were carried out in accordance with the National Commission for the Protection of Subjects of Biomedical and Behavioral Research guidelines for human studies and animal experiments. Before the study was initiated, written informed consent was acquired from all patients and approval was obtained from the local Ethics Committee of Sun Yat-Sen University (IRB number 201010).

### Patient Selection

Patients with congenital cholesteatoma and acquired cholesteatoma who underwent surgerybetween January 2011 and May 2014 in the Department of Otolaryngology Head and Neck Surgery, Sun Yat-Sen Memorial Hospital, were includedin this study. The inclusion criteria were as follows: 1) clinical signs and symptoms, radiological findings, and pathology consistent with cholesteatoma; 2) for congenital cholesteatoma, an intact tympanic membrane and no history of otorrhea; and 3)for acquired cholesteatoma, positive middle ear swabs during preoperative bacteriological examination. Patients who met the above criteria were enrolled in our study.

### Analysis of Clinical Aspects

Basic demographic information regardingthe duration of disease and the degrees of invasion of cholesteatoma, bone destruction, and hearing loss was collected and analyzed as previously described^46^. Briefly, the location of cholesteatoma was recorded according to surgical findings and included localization of the epitympanum, mesotympanum, aditus ad antrum, andmastoid antrum.The degree of invasion was classified into four grades: grade 1, involving one area; grade 2, two areas; grade 3, three areas; and grade 4, four areas. The degree of bone destruction was also classified based on surgical findings as follows: mild destruction, imperceptible erosion of the scutum and ossicle; moderate destruction, tegmen destruction and damage to most of the ossicle; and severe destruction, complete destruction of the ossicle and destruction of the bony labyrinth, the posterior wall of the external ear, and the facial canal. The degree of conductive hearing loss was determined by measuring the average air-conducted pure-tone auditory thresholds at 250, 1000, 2000, and 4000Hz and was classified as follows: mild, 26–40 dB; moderate, 41–55 dB; moderately severe, 56–70 dB; and severe, 71–90 dB. Detailed demographic informationis provided in Table 4.

### Tissue Collection

During each operation, the cholesteatoma was exposed via either tympanotomy or mastoidectomy. Congenital cholesteatomas and acquired cholesteatomas were obtained intraoperatively and placed in a 2-ml Eppendorf tube. Samples were stored in a HetoUltra Freezer (Thermo, USA) for further use in later experiments. If the samples were sufficiently large, some specimens were used more than once and were cut into three pieces to perform real-timePCR, Western blotting, and immunohistochemistry (IHC). Specimens from acquired cholesteatomas were matched to congenital cholesteatomaswith respect to age, sex, and hearing loss.

### Mice

Eight-week-old female C57BL/6 (B6) mice were purchased from Sun Yat-Sen University Animal Supply Center. TLR2−/− B6 and TLR4−/− B6 mice were originally generated by Shizuo Akira (Osaka University, Osaka, Japan). Additionally, the enrolled mice had to meet the following criteria: (1) the tympanic membrane was normal based on an otoscopic examination, and (2) the ABR thresholds were at normal levels (mean decibelsound pressure levels: <55 dBfor click stimuli, 40 dBfor 8-kHz stimuli,and 35 dB for 16-kHzstimuli). All mice were raised at the animal care facility at Sun Yat-Sen University Animal Supply Center. All animal experiments were performed in accordance with the National Institutes of Health Guide for the Care and Use of Laboratory Animals with the approval of the Scientific Investigation Board of Sun Yat-Sen University.

### Experimental Models of Acquired Cholesteatoma

Experimental models of acquired cholesteatoma have been previously described^47^. Briefly, the right tympanic membrane was laterally incised at the pars tensa with a sterile 25-gauge needle, andthe free margin of the tympanic membrane was then rolled into the tympanic cavity. A piece of autologous metal skin graft of approximately 0.5 × 1 × 1 mm was inserted into the bullae. Next, animals received an intratympanic injection of a bacterial suspension (5 μl)containing 100 colony-forming units of *P. aeruginosa*/μl (American Type Culture Collection 19660, cytotoxic), which were prepared as described previously^13^. At six weeks postoperatively, animals underwent endoscopic and anatomical microscopic examinations, ABR threshold evaluations, and histological analysis to characterize the clinical features of the experimental cholesteatoma.The degree of bone destruction was scored based on surgical findings according to human acquired cholesteatoma, and was graded from 1–4 as follows: 1, imperceptible erosion of the scutum and ossicle; 2,destruction of the tegmen and damage toone of the ossicles; 3,destruction of the tegmen and damage to two of the ossicles; and 4, complete destruction of the ossicle and destruction of the bony labyrinth and the posterior wall of the external ear.

### ABR Thresholds

The ABR was measured in each mouse prior to the operation and at six weeks postoperatively. The ABR thresholds of the mice were tested using a computer-aided evoked potential system, as previously described^28^. Click, 8-kHz, and 16-kHz pure-tone burst stimuli were delivered to the right ears of mice in a sound-isolated and electrically shielded booth (Acoustic Systems, Austin, TX, USA) via a Beyer earphone. Subdermal needle electrodes were inserted into the vertex and under both ears as ground electrodes.A signal-processing system (Tucker Davis Technology, Alachua, FL, USA) with SigGen/BioSigsoftware was used to generate stimuli and record responses. Thresholds were defined as the stimulus intensity between the minimum level that evoked the smallest ABR in wave V and the maximum level that produced no response. Based on the criteria of Zheng, average ABR threshold values of >55 dB (for click stimuli),40 dB (for 8-kHz stimuli), and 35 dB (for 16-kHz stimuli) indicated hearing impairment.

### Histological Analysis

Paraffin-embedded specimens were cut into 5-μm-thick sections, mounted on glass slides, and visualized using hematoxylin and eosin staining. Two pathologists separately evaluated the histopathological profiles of the specimens.

### RNA Isolation and Quantitative Real-Time PCR

Specimens were homogenized in TRIzol (Invitrogen), and RNA was extracted according to the manufacturer’s instructions. Spectrophotometry (260 nm) was used for quantification, and the 260/280-nm ratio was between 1.8 and 2.0.A 1-μg total RNA sample was reverse-transcribed into cDNA, and 2 μl of cDNA (1:10 diluted) was amplified in a 20-μl PCR reaction using SYBR Green Master Mix (Bio-Rad, Hercules, CA, USA). The primer sequences used to amplifyTLR2, TLR4, TLR5, TLR9, TNF-α, IL-1β, IL-10, inducible nitric oxide synthase (iNOS), BD-2, TRAP, RANKL, and osteoprotegerin (OPG)are shown in Table 5. Quantitative real-time RT-PCR reactions were performed using the CFX96 Real-Time PCR System (Bio-Rad), and relative mRNA levels were calculated after normalization to the levels of β-actin.

### Western Blotting

Specimens from each group were immersed in lysis buffer (2% sodium dodecyl sulfate (SDS), 10% glycerol, and 5% mercaptoethanol) and lysed using TissueLyser II (Retsch, Germany). Cellular debris was centrifuged at 10,000x*g* for 10 min, and the protein concentration in the supernatant was tested using a Quick Start Bradford protein assay (Bio-Rad). In total, a 20-μg protein sample was loaded into each lane of a 10% SDS-polyacrylamide gel electrophoresis (PAGE) gel, separated, and thentransferred to a supported polyvinylidene fluoride (PVDF) membrane (Bio-Rad). After blocking, blots were incubated with primary rabbit anti-human TLR2(Abs; 0.4 μg/ml; Cell Signaling), TLR4 (Abs; 0.2 μg/ml; Imgenex),TLR5(Abs; 0.2 μg/ml; Santa Cruz), and TLR9 (Abs; 0.4 μg/ml; Imgenex)antibodies at 4 °C overnight. Subsequently, blots were washed three times with PBST or PBS for 5 min and incubated with a secondary goat anti-rabbit IgG (H + L) Ab (1:5,000 dilution; LI-COR Biosciences, Lincoln, NE, USA) for 1 h at room temperature. Blots were then washed and detected using an Odyssey Infrared Imaging System (LI-COR Biosciences) according to the manufacturer’s instructions. Western blot band intensities were quantified using Image-Pro Plus 6.0 software.

### Immunohistochemistry

Paraffin-embedded sections were stained using immunoperoxidase staining techniques. Slides were deparaffinized in xylene, dehydrated in graded ethanol, and transferred to plastic Coplin jars with retrieval buffer (10 mM sodium citrate buffer, pH 6.0). Next, slides were heated in a constant-temperature tank for 20 min at 100 °C and allowed to cool at room temperature, followed by immersion in 0.3% hydrogen peroxide to block endogenous peroxidase activity. Immunoenzyme staining was performed using the biotin/streptavidin/peroxidase method with an anti-TLR4 Ab (1:50 dilution; Imgenex). The specificity of the Ab was verified by replacing the anti-TLR4 Ab with an isotype IgG control. Sections were assessed under a light microscope, and the number of TLR4-positive cells was quantified by image-enhanced histomorphometry, as previously described.

### TRAP Staining and Cell Counts

At 6 weeks postoperatively, temporalbones of the mice were collected for TRAPstaining. Paraffin-embedded temporal bone sections were washed twice with pre-warmed double-distilled water (37 °C), fixed with stationary liquid for 20 sec, and stained for TRAP (Sigma, St. Louis, MO,USA) for 60 min at 37 °C. Next, sections were gently washed again and counter-stained with hematoxylin in PBS containing 0.1% Triton X-100 at room temperature in the dark for 15 min. Sections were examined by light microscopy (Carl Zeiss, Oberkochen, Germany). Only cells with 3 nuclei were considered to be osteoclasts.

### Quantification of Viable Bacteria in the Middle Ear

Bacteria were quantitated in the infected middle ear of wild-type (WT),TLR2-deficient C57BL/6,and TLR4-deficient C57BL/6 mice at 3 and7 days postoperatively (n = 5 per group per time point). Middle ear effusions from infected ears were collected and individually washed 3 times with 5 μl of sterile 0.9% saline containing 0.25% BSA. Serial 10-fold dilutions of samples were plated on *Pseudomonas* isolation agar (Difco, Detroit, MI, USA) in triplicate, and plates were incubated overnight at 37 °C. The number of viable bacteria in the bullae was determined by counting individual colonies on each plate. The results are reported as the log_10_-transformed number of colony-forming units (CFUs) per ear ± SEM.

### Scoring system for middle ear pathology

A scoring system of −/+/++/+++ was used to evaluate the severity of pathology in the middle ears, as has been reported in otitis media studies^48^. Briefly, parameters were assigned as follows: −, absence of pathology; +, very scarce pathology in the middle ear; ++, pathology prevalent but not spanning the entire middle ear; and +++, pathology spanning the entire middle ear. Pathology was defined as inflammatory cell infiltration, tissue proliferation, and tissue debris.One point was assigned for each +, resulting ina maximum possible score of 9 points per mouse.

### Statistical Analyses

Statistical analyses were performed using SPSS for Windows version 16.0 (SPSS,Chicago, IL, USA).The mRNA expression levels of TLRs and inflammatory cytokines and the protein levels of TLRs in human congenital and acquired diseaseswere compared using Student’s *t*-test.One-way analysis of variance (ANOVA) was used to evaluate differences inthe mRNA expression levels of inflammatory cytokines, viable bacterial counts, and bone destruction scores in the mice.Data were considered to be statistically significantly different at P < 0.05. If a significant difference was detected, a Bonferroni test was used to assess differences between each of the two groups,whereas the relationship between the TLR4-positive cell counts and the clinical aspects was analyzed using the chi-square test. P < 0.05indicates that TLR4+ counts were related to the clinical criteria,while P > 0.05indicates that the counts were not related to the clinical criteria.

## Additional Information

**How to cite this article**: Si, Y. *et al*. TLR4 drives the pathogenesis of acquired cholesteatoma by promoting local inflammation and bone destruction. *Sci. Rep*. **5**, 16683; doi: 10.1038/srep16683 (2015).

## Figures and Tables

**Table 1:** Correlation of TLR4 Expressing cell Counts with Clinical Aspects in 187 Cases of Patients Acquired Cholesteatoma.

TLR4^+^cell counts	≤40	40–60	>60	*P value*
Age (years)				>*0.05*
≤45	42	25	23	
>45	35	33	29	
Sex				>*0.05*
Female	54	43	40	
Male	23	15	13	
Duration (years)				>*0.05*
≤5	54	43	40	
>5	24	14	12	
Invasion				<*0.05*
I	15	4	1	
II	24	4	1	
III	35	33	30	
IV	2	17	21	
Bone destruction				<*0.05*
Mild	48	11	2	
Moderate	19	17	13	
Moderate severe	3	21	23	
Severe	2	8	20	
Hearing loss				<*0.05*
Mild	30	19	15	
Moderate	29	20	18	
Severe	14	20	22	

**Table 2:** Semiquantitative evaluation of the pathology in middle ear 6 weeks postoperative.

Group	Click stimuli (M ± SD) dB	8 KHZ (M ± SD) dB	16 KHZ (M ± SD) dB
WT (preoperatively)	28.8 ± 13.2	26.3 ± 11.2	32.5 ± 13.2
TL2−/−(preoperatively)	25.4 ± 10.9	27.5 ± 16.5	30.2 ± 12.7
TLR4−/−(preoperatively)	32 ± 10.4	27.5 ± 9.6	25.0 ± 7.1
WT (6 weeks postoperatively)	57.5 ± 8.7	75.0 ± 9.1	67.0 ± 17.3
TLR4−/−(6 weeks postoperatively)	51.3 ± 7.8	73.0 ± 12.6	69.5 ± 18.5
TLR4−/−(6 weeks postoperatively)	35 ± 17.1	36.0 ± 16.7	36.8 ± 15.6

**Table 3:** Mean ABR thresholds of 8 cases of WT and TLR4−/− mice pre-and postoperatively.

Mice	Degree of pathological alteration in^b^			*Total scores*
	Inflammatory cells	Tissue proliferation	Tissue debris	
WT	2.75 ± 0.46	2.50 ± 0.50	2.50 ± 0.71	7.75 ± 0.13
TLR2−/−	2.38 ± 0.52	2.50 ± 0.53	2.00 ± 0.51	6.86 ± 0.10
TLR4−/−	1.75 ± 0.71	2.00 ± 0.76	1.38 ± 0.48	5.13 ± 0.14

**Table 4:** Demographic Data of Inclusion Patients.

	Sex		Media age (years)	Degree hearing loss				
	Female	Male		Normal	Mild	Moderate	Moderate sever	sever
Congenital Cholesteatoma	9	7	34(16–56)	7	4	3	1	1
Acquired Cholesteatoma	103	100	45(16–65)	0	65	53	51	34

**Table 5:** Sequences of Primers used in the PCR.

Gene	Oligonucleotide sequence (5′-3′)
Human β-actin (F)	GCTCCTCCTGAGCGCAAG
Human β-actin (R)	CATCTGCTGGAAGGTGGACA
Human TLR2(F)	CCGTAGATGAAGTCAGCTCACCGATG
Human TLR2(R)	CCTCCGACAGTTCCAAGA TGT AACGC
Human TLR4(F)	GAGGACCGACACCAATGATG
Human TLR4(R)	GAACGAATGGAATGTGCAACACC
Human TLR5(F)	TGCTCAAACACCTGGATGCTCACTAC
Human TLR5(R)	ACAGCCGCCTGGATGTTGGAGATATG
Human TLR9(F)	ACCTTCCATCACCTGAGCCATCTG
Human TLR9(R)	GCCGCTGAAGTCAAG AAACCTCAC
Human TNF-α (F)	CCAGGCAGTCAGATCATCTTC
Human TNF-α (R)	GTTATCTCTCAGCTCCACGC
Human IL-1β (F)	CGCAGCAGCACATCAACAAGAGC
Human IL-1β (R)	TGTCCTCATCCTGGAAGGTCCACG
Mouse β-actin (F)	GAT TAC TGC TCT GGC TCC TAG C
Mouse β-actin (R)	GAC TCA TCG TAC TCC TGC TTG C
Mouse TNF-α (F)	CACAGAAAGCATGATCCGCGAC
Mouse TNF-α (R)	TGCCACAAGCAGGAATGAGAAGAG
Mouse IL-1β (F)	CGC AGC AGC ACA TCA ACA AGA GC
Mouse IL-1β (R)	TGT CCT CAT CCT GGA AGG TCC ACG
Mouse IL-10(F)	AGCTGGACAACATACTGCTAACCGAC
Mouse IL-10(R)	CTTGATTTCTGGGCCATGCTTCTCTG
Mouse iNOS (F)	CTA AGA GTC ACC AAA ATG GCT CCC
Mouse iNOS (R)	AGA CCA GAG GCA GCA CAT CAA AGC
Mouse BD-2(F)	TCT CTG CTC TCT GCT GCT GAT ATG C
Mouse BD-2(R)	AGG ACA AAT GGC TCT GAC ACA GTA CC
Mouse-TRAP (F)	CGC-ACA-GGT-AGGCAG-TGA-C-
Mouse-TRAP (R)	CTA-CCC-CGT-GTGGTC-CAT-AG-
Mouse-RANKL (F)	TAACCCTTAGTTTTCCGTTGC
Mouse-RANKL (R)	CCTGAGACTCCATGAAAACGC
Mouse-OPG (F)	TCCTGGCACCTACCTAAAACAGCA
Mouse-OPG (R)	CTACACTCTCGGCATTCACTTTGG
